# Use of integrated services in antenatal care: A case study of Mabvuku Polyclinic, Zimbabwe

**DOI:** 10.4102/phcfm.v17i1.4847

**Published:** 2025-05-30

**Authors:** Gamuchirai P. Gwaza, Danai T. Zhou, Annette Plüddemann, Carl Heneghan

**Affiliations:** 1 Nuffield Department of Primary Care Health Sciences, University of Oxford, Oxford, United Kingdom; 2 Department of Laboratory Diagnostic and Investigative Sciences, University of Zimbabwe, Harare, Zimbabwe

**Keywords:** integrated diagnostics, maternal healthcare, healthcare workers, antenatal care, Zimbabwe

## Abstract

**Background:**

The integration of diagnostic services presents a critical opportunity to improve health outcomes in low- and middle-income countries (LMICs), potentially averting up to 1 million premature deaths annually. Antenatal care provides a critical platform for diagnosing multiple diseases in an integrated manner.

**Aim:**

This study explored the experiences of healthcare providers and pregnant women using integrated diagnostic services at a primary care facility in Zimbabwe.

**Setting:**

A qualitative case study was conducted at Mabvuku Polyclinic in Harare, Zimbabwe.

**Methods:**

Using purposive sampling, 14 healthcare workers and 22 pregnant women participated in interviews. Observations and semi-structured interviews were recorded, transcribed and analysed using NVivo software. Thematic analysis was applied to identify key themes related to access, patient-provider interactions and systemic barriers.

**Results:**

According to the interviewees’ reports, challenges such as limited resources, medical equipment and staff hinder efforts to integrate diagnostic services. The women strongly preferred integrated diagnosis, even if it meant enduring long waiting times, and valued the convenience of receiving all necessary services in a single visit. The study highlighted the hidden socio-economic barriers to ‘free’ healthcare and underscored the importance of addressing systemic inefficiencies.

**Conclusion:**

The insights gained from this study are transferable and contribute to the understanding of integrated diagnostic services in maternal healthcare contexts.

**Contribution:**

They offer practical recommendations for improving service delivery and health outcomes in similar settings.

## Introduction

Addressing diagnostic gaps for key conditions such as diabetes, hypertension, human immunodeficiency virus (HIV), tuberculosis (TB), hepatitis B virus infection, and syphilis could avert approximately 1 million premature deaths annually in low- and middle-income countries (LMICs).^1^ In Zimbabwe, maternal health services integrate diagnosis for these conditions during antenatal care visits, ideally conducted within the first 12 weeks of pregnancy.^2^ This aligns with the World Health Organization (WHO) recommendation for diagnosing these six tracer conditions critical to maternal and child health.^1^

Integrated diagnosis transforms health systems into patient-centric structures, moving beyond a disease-centric approach.^3^ Integrated diagnosis functions as a specialised component within the broader framework of integrated healthcare, concentrating on a specific stage in the care cascade.^3^

This study explored the experiences of healthcare workers (HCWs) and pregnant women in the community who utilise integrated diagnostic services at a primary health care facility. The results generated insights and evidence to inform the design of integrated diagnostic interventions and maternal health services in similar low-resource contexts.

## Background

### Zimbabwe’s healthcare system

Zimbabwe’s health service delivery system operates at four levels: primary, secondary, tertiary and quaternary. Primary health care is the backbone, offering a range of services, including maternal and child health services, health education, immunisations, infectious disease control and basic preventive and curative services.^4^ Health services are integrated at the service Mabvuku Polyclinic’s maternity services offering curative and preventive services, including maternal and child health and family planning.

The public sector, led by the Ministry of Health and Childcare (MOHCC), is the primary health care provider nationwide, supplemented by the private sector, which includes for-profit hospitals, maternity homes and not-for-profit mission clinics.^4^ Zimbabwe has 10 provinces, with Harare and Bulawayo functioning as cities with provincial status. In Harare, healthcare facilities, such as clinics and Polyclinics, are managed by the Harare City Council (HCC), while the MOHCC oversees tertiary and quaternary care facilities (advanced specialised care). Mabvuku Polyclinic operates within this framework under the HCC.

### Maternal health services in Zimbabwe

Maternal and neonatal health services are priorities in Zimbabwe’s healthcare system,^4^ supported by various donors. Despite progress, access remains a significant challenge, particularly for economically disadvantaged women. To address this, Zimbabwe adopted a ‘supermarket approach’,^4^ integrating maternal and neonatal health services into single facilities or co-located settings, ensuring convenience and accessibility.^3^

Maternal mortality has declined from 570 per 100 000 live births in 2010 to 357 per 100 000 live births in 2020^4,5^ but remains high, underscoring the need for continued improvements. Barriers to adequate antenatal care must be addressed to sustain progress. In child health, the under-5 mortality rate decreased from 69 deaths per 1000 live births to 37 deaths per 1000 live births in 2020, with 70% of deaths occurring during infancy.^6^ The neonatal mortality rate, at 29 per 1000 live births, accounts for 40% of childhood deaths,^7^ emphasising the importance of targeted interventions during the pre- and post-natal periods.

While 94% of the pregnant women attend at least one antenatal visit, only 76% receive ‘adequate’ antenatal care (four visits).^4^ Among those, 98% had blood samples taken, 97% had blood pressure (BP) measured and 68% had urine samples taken.^7^ However, only 54% received sufficient tetanus toxoid injections.^7^ These statistics show the need to improve the quality and accessibility of maternal healthcare services to ensure better outcomes for both mothers and their infants.

### Integrated diagnosis services

Diagnosis is critical in improving overall health system performance,^1^ yet gaps in adherence to recommended testing protocols persist, particularly in Zimbabwe’s antenatal care. The standard antenatal care protocol recommends a series of laboratory tests during the first 12 weeks of pregnancy,^2^ including HIV testing (with CD4 count or viral load for those already positive), syphilis screening, TB and malaria tests (when presumed) and a full blood count (FBC), among others. The integrated diagnosis model aims to conduct all these tests during a single visit and provide results on the same day.

However, HCWs often fail to consistently follow the prescribed testing protocol. Essential tests such as hepatitis B screening, blood grouping and ultrasound scans are sometimes overlooked.^8^ This inconsistency in adherence limits early detection of potential health issues, leading to missed opportunities to manage potential complications during pregnancy.

### Study rationale

While much has been studied about patient experiences in integrated healthcare,^6^ fewer studies have focused on integrated diagnosis.^9^ Given that the primary objective of integrated healthcare is to improve patient experiences and health outcomes,^10,11^ it is essential to examine how these interventions work and can be optimised. A patient-centred approach, which considers the perspectives of HCWs and patients, is key to designing effective healthcare solutions.^12^

Maternal health provides an ideal framework for integration in LMICs, as pregnant women have regular contact with the healthcare system.^13^ This regular contact offers a valuable opportunity to integrate additional services. Insights from maternal health integration can inform broader healthcare strategies, making it an important case study for improving overall health service delivery.

## Research methods and design

### Study design

This is a qualitative study using a case study methodology rooted in postpositivist critical realist thinking, which focuses on understanding individual experiences to gain insights into external reality.^14^ The study also applied human-centred design thinking to design interview questions, focusing on ideal scenarios and future models for intervention design. This meant participants were asked about their experiences as well as the ideal scenario for them, ensuring an empathetic, bottom-up strategy that includes participants perspectives to improve intervention delivery.^12^ The study aimed to explore the experiences of HCWs and pregnant women in accessing integrated diagnosis services within the context of antenatal care at Mabvuku Polyclinic.

### Study setting

Mabvuku Polyclinic, located 22 km from Harare’s central business district (CBD), serves over 500 000 people, including residents of expanding high-density suburbs, such as Caledonia and Bobo Farms.^15^ It offers various services, including general outpatient care, maternal and child health services, HIV counselling and testing, TB care and laboratory services.^15^ In 2016, the clinic’s maternal health unit was upgraded to provide caesarean sections, reducing referrals to secondary and central hospitals.

Before 2015, the clinic conducted about 3000 antenatal checks and 1000 deliveries annually.^15^ Following the upgrade, this increased to 5000 antenatal checks and 3000 deliveries, with a 40% reduction in referrals to Sally Mugabe Central Hospital.^15^ However, all complicated deliveries are still referred to the central hospital, and many women with anticipated complications bypass Mabvuku Polyclinic’s antenatal care services, opting for direct care at Sally Mugabe Central Hospital.

### Study population and sampling strategy

Two groups of people were targeted: healthcare providers and pregnant women accessing antenatal services.

### Inclusion and exclusion criteria

Healthcare workers: Inclusion criteria included being 18 or older, qualified as midwives, nurses, laboratory technicians, or doctors and providing informed consent.

The inclusion criteria for the participating women required a confirmed pregnancy, being 18 years or older, actively receiving antenatal care at the clinic and providing informed consent. There were no restrictions based on literacy level, occupation or other socio-demographic characteristics.

### Sampling strategy

Healthcare workers were purposively sampled for maximum variation and to ensure targeted insights were gathered to ‘saturation’,^16^ based on roles and availability, with help from facility officials and the HCC Department of Health. Snowball sampling techniques were also used to seek referrals.

The study was conducted over a 2-week period, during which pregnant women attended their first antenatal visit on Mondays, Tuesdays and Wednesdays – three times a week. On average, 30–50 women attended each session, with an estimated 35 women per day. Over the 6 clinic days, at least 120 pregnant women were seen.

Participating women were recruited through health education announcements and systematically selected from those exiting the facility. To ensure randomisation, every third pregnant woman meeting the eligibility criteria was invited to participate. Eligibility was determined by asking whether the woman had received antenatal care services and secured a booking, as only confirmed pregnancies were officially booked. If a selected woman did not qualify, the next eligible participant was invited to maintain the sample size.

Given that each interview lasted between 20 and 40 min, a steady flow of women exited the facility during the study period. Because of time constraints and the presence of only 1 researcher, the target was to interview 20 women; however, a total of 22 women were ultimately interviewed.

Participants were briefed on the study’s purpose, and informed consent was obtained via signed forms in Shona or English.

### Data collection

Two main data collection tools were used: an interview guide for HCWs and another for pregnant women. The questions were developed based on core criteria for integrated diagnostic interventions, designed using a Delphi study (Gwaza, 2023).

The interview guide for pregnant women focused on access to services, timeliness of care, relationships with HCWs and challenges and recommendations for service delivery. To ensure clarity, the questions were piloted with two community members outside the facility.

The interview guide for HCWs was structured around the six WHO health system pillars: leadership and governance, service delivery, health workforce, information systems, health commodities and financing. These questions were translated into Shona, the local language, with the assistance of a local researcher.

In addition, a structured observation checklist was used to assess key service delivery metrics, including waiting times, ease of patient access, patient pathways, HCW behaviour and general facility processes, such as the registration process.

#### Semi-structured interviews

These were conducted with HCWs and pregnant women using tailored guides. At least 30 interviews were planned: 10 with HCWs and 20 with pregnant women. Totally, 32 interviews were conducted (10 HCWs and 22 pregnant women). The interviews lasted 20–30 min and were conducted face-to-face in Shona or English.

#### Structured non-participant observation

Two weeks were spent observing antenatal visits, registration processes, waiting dynamics and reporting tools. A checklist guided the observations, and building rapport minimised the observer effects. The observation data were triangulated with interview findings to assess alignment. For example, patient-reported experiences were compared with observed waiting times to identify consistencies or discrepancies.

### Recruitment

#### Data management and analysis

Interviews were audio-recorded and transcribed orthographically, preserving speech patterns like hesitations, pauses and emphases. Non-essential words were removed for clarity while maintaining meaning.^17^ Personal identifiers were excluded except for relevant organisational references. Transcripts were anonymised and assigned numerical codes (e.g., Participant 1–22 and healthcare worker as HCW 1–10). Accuracy and completeness were rigorously checked.

The audio files and transcriptions were securely stored on password-protected OneDrive for Business within Oxford University’s Nexus365 platform, while physical consent forms were kept in locked cabinets. Data retention follows university policy, ensuring storage for at least 3 years post-publication.

Qualitative data analysis was performed using NVivo (Version 14) software, employing thematic analysis to identify patterns across the data set. This method, suitable for interpreting qualitative data, used a primarily deductive approach, deriving initial codes and themes from pre-existing concepts.^17^ Emergent themes were also incorporated.

### Ethical considerations

Ethical clearance to conduct this study was obtained from the University of Oxford Tropical Research Ethics Committee (No. 520-24), and permission to access participant information was secured from the City of Harare Director of Health Services dated 27 February 2024. The study posed a minimal risk, with no expected harm beyond daily life or routine tests. After their involvement, pregnant women received transport reimbursement. No medical records, sensitive health data or patient consultations were reviewed; all data were self-reported. All participants provided written informed consent before participation.

## Results

### Demographic details

As many as 22 pregnant women attending antenatal care and 10 HCWs were interviewed. Among the women, 14 attended their first visit, 3 their second, 4 their third and 1 her fourth. Ages ranged from 18 to 36 years, with an average age of 27 and two children each. Most^16^, (*n* = 16) had completed their ordinary-level education, while two had higher education and were employed as teachers. However, 13 were unemployed (Table 1).

**TABLE 1:** Demographic details of the 22 community women.

Age (years)	Frequencies
18	1
19	2
20	3
22	2
23	4
27	1
29	2
30	1
32	1
33	1
34	1
35	1
36	2
**Profession**	
Carpenter	1
Cleaner	1
Hairdresser	1
Security guard	1
Teacher	2
Unemployed	13
Vendor	3
**Highest level of qualification**	
Primary (Grade 7)	1
Form 2	1
Form 3	1
Form 4	16
Bachelor’s degree	2
Master’s degree	1
**Number of children**	
0	7
1	8
2	3
3	3
4	1
**Type of visit**	
1st visit	14
2nd visit	3
3rd visit	4
4th visit	1
**Mode of transport to the facility?**	
Kombi (public taxis)	16
Walked	5
Drove	1

The 10 HCWs included 2 laboratory technicians, 2 primary care counsellors and 6 midwifery’ nurses. Three midwives held leadership positions: sister-in-charge, district nursing officer and acting matron. Four midwives had over 15 years of experience at the facility, and two had 9. One laboratory technician and primary care counsellor had 1 year of experience at the facility, while their counterparts had over 5. Only one HCW was male.

### Description of the patient journey

**FIGURE 1:**
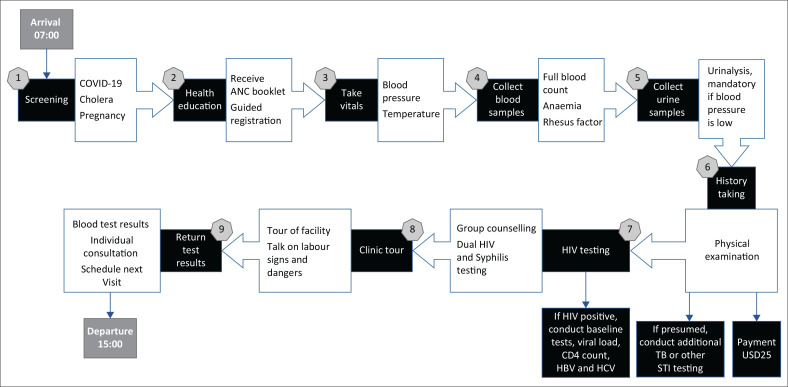
Pathway for the community women at Mabvuku Polyclinic attending the first antenatal care or new booking.

Interviews with the pregnant women occurred as they exited the facility or waited for the test results, typically after 14:00. Women underwent an average of nine care stages, each lasting 10–30 min. Additional steps are applied to those with presumed conditions or HIV. Figure 1 outlines the patient journey stages.

Participants reported waiting outside the clinic upon arrival, spending an average of 8–10 h at the facility. Based on observations and interviews, most women arrived between 05:00 and 07:30, with departures varying from 15:00 to 17:00, depending on the services received and personal circumstances (Table 2).

**TABLE 2:** Arrival time at the facility.

Approximate time of arrival	05:00	06:00	06:30	06:45	07:00	07:30	08:00	08:30	08:40
Number of women	1	2	2	1	4	4	6	1	1

Women were served based on arrival time; no formal numbering system was used. Coronavirus disease 2019 (COVID-19) and cholera screenings were reportedly not conducted during the study, although 14% of the participants received face masks. By 08:00, women moved from outside benches to those within the clinic for health education sessions led by midwives. New bookings occurred from Monday to Wednesday, while repeat visits were on Thursdays and Fridays. Services were delivered in batches without individual numbering. Student nurses recorded vital signs (BP and temperature), and antenatal booklets were provided for demographic information.

Midwives conducted consultations to assess medical history and symptoms. Confirmed pregnancies required a booking fee (USD 25) for further services, including blood tests for FBC, blood grouping, and urinalysis, followed by tetanus injections, group HIV counselling and individual testing, HIV seropositive women were referred for treatment, with HIV viral load testing conducted if necessary. Women with a history of complicated pregnancies were referred to the Sally Mugabe central hospital. Women received a facility tour and health talks and reviewed results with midwives after a 4-h wait. Scans were available for USD 10.

Once the results were available, each woman consulted individually with a midwife to review findings and receive prescriptions such as iron supplements for anaemia. All participants were scheduled for their next antenatal checkup. Women accompanied by spouses, as with Participant 18, were prioritised to encourage male involvement in antenatal care and HIV and syphilis testing. Participants reported being served by at least two nurses.

### Themes identified

Six themes were identified in the study – three from the pregnant women’s perspectives and three from the HCWs’ perspectives. A summary of the themes is provided in Table 3.

**TABLE 3:** Summary of the main themes identified.

Target group	Themes identified
Pregnant women	Preference for integrated diagnosis despite long waiting times Integrated diagnosis Waiting time The participants considered respectful and dignified treatment by HCWs as a key measure of the quality of care. Positive relationship between pregnant women and HCWs Physical environment Linkage to follow-up care There are hidden costs to integrated diagnosis Cost of services Hunger Discomfort and physical strain
Healthcare workers	4. The ability to provide optimal care was hindered by a lack of resources and tools Staff shortages Lack of materials 5. Effective diagnosis requires a complete continuum of care – testing, treatment and follow-up. 6. Fragmented donor support and inconsistent funding have disrupted effective service delivery, creating resource gaps and undermining care continuity.

#### Preference for integrated diagnosis despite long waiting times

##### Integrated diagnosis

Mabvuku Polyclinic’s antenatal clinic offered comprehensive, integrated diagnostic services, including FBCs, anaemia screening and syphilis testing. Tests for the Rhesus factor, crucial for preventing complications like hydrops fetalis and stillbirths, were unavailable during the study due to material shortages, although such issues are rare among African populations.^18^

Syphilis and HIV tests were conducted using dual rapid test kits managed by primary care counsellors. Positive cases were referred to midwives, with HIV-positive women receiving further tests like viral load, CD4 counts and hepatitis screenings. TB testing was provided for women with low CD4 counts. On-site laboratory machines supported diagnostics for TB, HIV and other conditions, including simultaneous testing for TB, HIV viral loads, early infant diagnosis (EID) for HIV and COVID-19.

Integrated diagnosis of HIV and syphilis was carried out even without the dual test. According to HCW 1, when the dual HIV and syphilis test kits ran out at Mabvuku Polyclinic, syphilis testing was continued separately in the laboratory. Primary care counsellors, primarily funded by the Global Fund for HIV testing, adapted to perform syphilis tests due to the dual functionality of the kits. Once the syphilis test kits were depleted, the counsellors focused solely on HIV testing. Women who tested positive for either HIV or syphilis were encouraged to invite their spouses for testing. However, some spouses were resistant, leading counsellors to collect contact details for follow-up during the woman’s next antenatal visit. In addition to antenatal care, the clinic offered diagnostic services for TB, diabetes and sexually transmitted infections (STIs) like gonorrhoea. However, a shortage of glucometer strips during the research period meant that blood glucose testing was unavailable. These diagnostic services were part of the broader integrated healthcare approach, serving antenatal clients and the wider facility population.

Previously, delays in test processing, often lasting weeks, hindered timely care. On-site testing addressed these delays, ensuring faster results. However, at least three women reported delaying their first antenatal visits, missing opportunities for early diagnosis.

##### Waiting time

Much of the time spent at the Mabvuku Polyclinic was dedicated to waiting, particularly for blood test results. Women often waited between services, with the longest wait for test results averaging 4–6 h:

‘Whatever they did to me and however long it took, they need to do the same for 30 other women after me. It took me 30 minutes to finish the HIV test, and I had to wait for the others to receive the same test.’ (Participant 9, 36 years, self employed)

However, despite the extended wait, many women appreciated completing all services in 1 day. Participant 1 remarked, ‘It’s better to come and wait and have everything done in 1 day than to come back another day for results. It’s like killing many birds with one stone.’ (Participant 1, 30 years, teacher). *Eight of the women* agreed that it saved transportation costs and minimised the time taken off from work or childcare.

However, two participants expressed frustration with the long wait time. Participant 3, a teacher, shared that she was unprepared for the long delay. According to Participant 3:

‘It’s my first time, maybe because I wasn’t aware that it took such a long time when I came in the morning at 8 o’clock, I thought they would just check me, and I [*would*] leave. But they had to take my blood and send it to the lab, and we had to wait for the results, which were delayed and only came out after 1 pm. So that was my main challenge because I was hungry and sitting on the bench for a long time was painful. The waiting part is a challenge, but maybe if I had known that I would have to wait, the experience might have been better.’ (Participant 3, 29 years, teacher)

Despite this, she recognised the overall benefit of completing all services in one visit.

The participants considered respectful and dignified treatment by HCWs as a key measure of the quality of care.

The quality of care highlights the overall care experience, emphasising key components such as the relationship between patients and HCWs, characterised by respect, kindness and clear communication. It also considers the facility’s physical environment, including privacy and the effectiveness of follow-up services. Timely delivery of results and providing clear guidance on the next steps in treatment further contribute to the quality of care.

### The participants considered respectful and dignified treatment by HCWs as a key measure of the quality of care

#### Positive relationship between pregnant women and healthcare workers

The pregnant women chose the Mabvuku Polyclinic for several reasons: positive staff attitudes, proximity to their homes and comprehensive care. Recommendations from friends and family influenced 10 women, while 6 women valued convenience and proximity, particularly in emergencies, although 3 planned to deliver at a private hospital. Participant 17 selected the clinic because it accepted first-time pregnancies unavailable elsewhere. Participant 13, who was visiting Mabvuku Polyclinic for the first time, had this to say:

‘I am very happy with the services here. It’s closer to my home, but I always thought the clinic would not offer many services. For my first pregnancy, I went to XX (a provincial hospital nearly 40 km from her home), but the nurses’ attitudes were terrible. I had to wake up at 05:00 to arrive by 07:00 to attend the antenatal visits. I wish I had known they had such a nice facility here where I could deliver my baby.’ (Participant 13, 36 years, unemployed)

**FIGURE 2:**
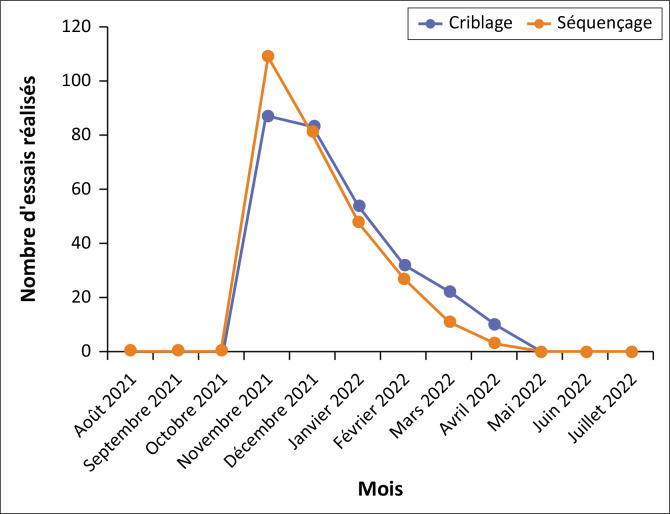
Participants describing their experiences with interaction with the health workers.

Regarding HCW attitudes, most participants (18 out of 22) reported positive experiences and feeling respected, dignified and cared for. The midwives, affectionately called ‘mbuya’ [grandmother], were praised for their motherly approach. Six participants appreciated their willingness to answer questions and offer guidance. Participant 13, for example, noted that a midwife encouraged her to ask questions for clarity. Participants felt well cared for, with no complaints about the staff’s attitudes (Figure 2).

Twelve participants reported a clear understanding of the procedures, with midwives providing detailed explanations before and after each step, including blood draws and tetanus injections. They appreciated the midwives’ efforts to explain the purpose of each action for their safety and that of their children. Participant 1 compared the experience favourably to her previous pregnancy at another facility, stating, ‘The way I was treated here is far much better.’ (Participant 1, 30 years, teacher)

However, two participants faced challenges with clarity. Participant 3 felt the midwives provided unclear explanations, merely directing her without context. ‘They were just saying come here, sit there and wait, get this injection.’ (Participant 3, 29 years, unemployed). Given the many people, she also expressed reluctance to ask questions, fearing it would slow the process. She said, ‘There are too many people. They just want you to follow instructions so the line can move faster.’

Overall, participants trusted the midwives’ expertise, relying on them to inform them of any issues. The trust was evident, as most participants^19^ recalled only key procedures such as the HIV test, urine test, blood draw and tetanus injection, while only four remembered the syphilis test. Two participants forgot certain tests but could refer to their booklets. Two noted that HCWs only explained positive results and the next steps. Four of them struggled to remember everything provided but trusted the HCWs to guide them on necessary steps, particularly regarding positive results. Participant 9 expressed this trust, saying, ‘I just trust that the nurses know what they are doing, and if there is a problem, they will tell me.’ (Participant 9, 36 years, self employed)

Despite overall positive feedback, four participants described feeling rushed, overwhelmed or confused by the pace or manner of treatment. They attributed these feelings to the high patient volume.

- Participant 17 noted, ‘Treatment was good, but they were rushing us a bit, which I can understand because we are too many people.’ (Participant 17, 20 years, unemployed)
- Participant 19 commented on the midwives’ expectations, ‘They just want you to be energetic, not dragging yourself or appearing weak. You have to make yourself strong.’ (Participant 19, 29 years, unemployed)
- Participant 3 described the need to stay alert: ‘They just urge you to be alert and know what’s happening around you … not really rude but firm.’ (Participant 3, 29 years, unemployed)
- Participant 9 acknowledged the challenges HCWs face with a high patient volume, contrasting the care with private facilities: ‘You cannot compare it with private care where there are fewer people.’ (Participant 9, 36 years, self employed)

Healthcare workers reported mixed experiences in engaging with the women. Healthcare workers 9 and 10 reported that some women hesitated to open up despite their efforts to create a supportive and non-judgemental environment. Some women, aware of their status but not adhering to their treatment at other clinics, feared stigma or judgement, delaying disclosure until after extended discussions.

The positive and friendly demeanour was also noted during observations, as midwives were seen offering advice to pregnant women while carrying out their duties and interacting with one another. Positive patient perceptions were attributed to several factors. Key among these was customer care training organised by the city council, involving all staff, including janitors and nurses. Healthcare workers 4, 5 and 6 noted that reminders about patient rights, reinforced in morning briefings and through visible information sheets and posters, shaped a patient-centred approach. These materials also included feedback mechanisms deemed effective by HCWs.

Healthcare worker 4 emphasised maintaining a friendly demeanour, saying, ‘I smile because it’s because of patients that I get the little salary I have. If you’re angry with the patient, you’re in the wrong profession.’ (HCW 4).

#### Physical environment

Participants appreciated the facility’s commitment to privacy and respect, especially during sensitive care stages. Critical procedures, including HIV testing, full-body exams, history-taking and result discussions, were conducted in private rooms, fostering a comfortable environment. Less sensitive tasks, such as blood draws and injections, occurred communally, typically involving three women at a time. This practice was observed and later confirmed during interviews.

Healthcare workers emphasised the importance of privacy, particularly during HIV and syphilis counselling sessions, which lasted 25–30 min each. These individual consultations ensured confidentiality and encouraged open discussions. Despite serving about 30 pregnant women per visit, the facility effectively managed privacy, prioritising respectful and secure care during key interactions.

#### Linkage to follow-up care

Treatment for most conditions, including HIV and syphilis, was generally available. However, the availability of iron supplements was inconsistent. Three participants reported receiving prescriptions but were uncertain about the availability or purchasing of these supplements. Healthcare workers 5 and 6 observed that many women returned for follow-up visits with persistently low haemoglobin levels, often due to the intermittent supply of iron supplements, limiting effective anaemia management.

### There are hidden costs to integrated diagnosis

Integrated diagnostic services often come with hidden costs that can burden the women. Long waiting times resulted in discomfort, such as hunger and back pain from prolonged sitting. Participants had to bear extra expenses, such as paying for scans.

#### Cost of services

The antenatal care fee at the polyclinic was USD 25, covering all visits until delivery and the baby’s first month of care. However, ultrasound scanning services require an additional USD 10, a significant cost for many participants from the low-income community. While some opted for central hospitals offering free maternal health services, they encountered mixed messaging. Although labelled ‘free’, participants reported paying extra to expedite care, with one noting, ‘It’s free, but you have to pay something’.

Over half of the participants^12^ were unemployed. Financial constraints meant some delayed care, as in Participant 18’s case, where she postponed her scan until she could raise funds. Three participants reported paying USD 5 extra to bypass daily limits, which HCWs could not confirm. The participants noted that such practices were subtle and not always apparent unless directly experienced.

#### Hunger

Hunger during the lengthy waiting process was a significant challenge for many participants (*n* = 9). Participants often brought their own food or purchased meals from vendors outside the facility. Participant 14 highlighted the unpredictability of the process duration as a key concern. Participant 18 noted being delayed after leaving to find food, which disrupted her appointment schedule. Similarly, Participant 20 explained that stepping out for food increased her risk of losing her place in line, prolonging the wait. Even bringing a lunchbox was not a complete solution, as leaving to eat outside posed the same risk. She suggested hiring additional staff to reduce waiting times.

#### Discomfort and physical strain

Participant 17 attributed her back pain to the prolonged waiting time and sitting on hard benches all day. She remarked, ‘They try to solve one problem but create many others. I’ll probably leave more sick than I came. They need more nurses for faster service or to allow new bookings any day of the week.’ (Participant 17, 20 years, unemployed). The researcher observed women frequently standing, stretching or walking to ease discomfort from back and leg pain.

### Experiences of the healthcare workers providing care

The ability to provide optimal care was hindered by a lack of resources and tools.

All the HCWs felt that their ability to provide integrated services was hindered by limited resources, particularly staff shortages and a lack of materials.

#### Staff shortages

Mabvuku Polyclinic operates with only half of the required staff, significantly straining its ability to provide integrated services. Healthcare workers reported handling 30–50 women daily, leading to long waiting times and rushed procedures. Staff shortages forced HCWs to skip steps during peak times, increasing risks like missing breach detections during palpation. Healthcare worker 7 described working through lunch to ensure timely results, while HCW 4 lamented the ‘pain’ of the overwhelming workload.

The scheduling system is intended to help with work planning. The Polyclinic’s schedule allocates the first 3 days for new bookings and the last 2 for repeat visits, but managing both groups’ differing needs remains challenging. For instance, Participant 21 came on the wrong day and faced delays, illustrating scheduling inefficiencies.

While the HCC leadership acknowledges the staffing crisis, immediate recruitment of midwives is not feasible. The clinic also relies on contract nurses from other hospitals, but HCWs noted these arrangements are temporary fixes that are insufficient to address systemic shortages effectively. Healthcare worker 2 criticised these contract/locum nurses as wasteful and not understanding the facility’s long-term needs.

Staff shortages were also linked to low morale and retention rates hindered by little and delayed salaries, and workers frequently sought better opportunities elsewhere. Healthcare worker 6 mentioned that she stayed because she had no other options or places to go. Healthcare workers 4, 5 and 6 also noted a lack of incentives, such as food packs, previously provided by organisations like the Red Cross.

According to HCW 6:

‘Last time, the Red Cross gave us food packs and $50, which at least boosted the morale of the staff. When you are hungry and have no food at home, and you don’t have the ZIG (Zimbabwean gold currency) , and we have not received our salaries from March. What do you do? You resort to abusing materials.’ (HCW 6)

#### Lack of materials

Healthcare workers 4, 5 and 6 identified severe material shortages that hindered the clinic’s operational efficiency. Essential items, including infant radiant warmers, glucometers, specimen jars, syringes and soap, were unavailable or dysfunctional. Healthcare workers 7 and 8 also pointed out frequent breakdowns of laboratory equipment, such as the FBC analysers, due to overuse, a lack of maintenance or missing cartridges.

The laboratory’s conditions also exacerbated equipment failure; excessive heat and unreliable electricity, which impacted machines like the biochemistry analyser, which remained non-functional for 2 years. Healthcare worker 7 expressed frustration, saying, ‘That machine has never worked because when it was installed, there was no electricity.’ (HCW 7)

Iron tablets were frequently unavailable, forcing HCWs to prescribe unaffordable alternatives, which led to persistent anaemia among pregnant women, confirmed by three participants. Healthcare workers also reported shortages of surgical materials, requiring patients to buy their own supplies. Critical test kits for HIV infection duration and Rhesus typing were also missing, complicating newborn testing in the labour ward.

Low salaries exacerbated issues, leading to theft and misuse of resources, as some staff sold medications or used them personally. Healthcare worker 4 lamented, ‘Low salaries lead to abuse… A cow must graze where she is tied.’ (HCW 4)

Donor dependency was highlighted, with HCWs suggesting charges for materials to ensure sustainability. Vandalised oxygen pipes, an unfuelled generator and a single functional BP machine symbolised the clinic’s resource constraints.

Despite its modern infrastructure, HCW 3 emphasised better maintenance and reliable supply chains to overcome Zimbabwe’s broader economic challenges and sustain operations effectively.

Effective diagnosis requires a complete continuum of care – testing, treatment and follow-up.

Limited resources, including the unavailability of anaemia treatments and logistical barriers, delay care and compromise patient outcomes.

The clinic, upgraded to perform elective caesarean sections, lacks a resident doctor and refers complicated pregnancies to hospitals. While caesarean sections are scheduled weekly, only one or none are performed monthly.^15^ Strict adherence to the MOHCC protocols, requiring referrals for conditions like pre-eclampsia or diabetes, even when manageable by nurses, further delays care. Healthcare workers 2 and 3 reported that nurses occasionally deliver babies with complications before ambulances arrive.

Referrals are hindered by ambulance accessibility. Private ambulances, costing USD 70–100, are unaffordable for most patients, while city ambulances, although more affordable and sometimes available on credit, are not consistently reliable. Participants expressed frustration, emphasising that emergencies should prioritise life over payments. Participant 19 noted, ‘The nurses tell us to look for money to pay for the ambulance when the focus should be on preserving life.’ (Participant 19, 29 years, unemployed)

Fragmented donor support and inconsistent funding have disrupted effective service delivery, creating resource gaps and undermining care continuity.

Multiple partners, including the Global Fund, AIDS Healthcare Foundation (AHF), Biomedical Research and Training Institute (BRTI), Cordaid, Red Cross and Zimbabwe Network for Health-Europe, fund different areas without cohesive integration. Healthcare workers 7 and 8 reported that while the Global Fund supports HIV primary care counsellors, AHF funds laboratory technicians, leaving gaps in critical areas like internet access. For example, laboratory data transmission remains limited despite primary care counsellors receiving data bundles, as these cannot be shared due to restricted allocations.

Unmet needs persist despite donor involvement. Healthcare worker 1 noted that equipment, such as the United Nations International Children’s Emergency Fund (UNICEF)-donated BP machines, sits unused due to fragmented supply chains. Healthcare workers 2 and 3 highlighted stock shortages, often receiving ‘out of stock’ responses for vital items with no clear inventory status at higher levels.

This fragmentation has exacerbated workload disparities. Donor-funded staff often handle more tasks than city council-funded counterparts, causing resentment. As HCW 7 remarked, ‘You do the work; you earn in USD’, reflecting frustration over inequitable pay structures.

Frequent staff absenteeism for donor workshops further disrupts operations. These workshops provide critical per diems for underpaid staff but leave the clinic understaffed. At the time of the study, the facility lacked substantive leadership, with only an acting matron. This leadership gap meant key issues were overlooked, diminishing care quality and efficiency.

## Discussion

Mabvuku Polyclinic, a recently upgraded Polyclinic, exemplifies the complexities of delivering effective maternal healthcare in resource-limited settings. Despite its improved infrastructure, significant barriers continue to hinder service delivery, reflecting common challenges faced in LMICs. The primary objective of this study was to explore the experiences of HCWs and pregnant women in accessing integrated diagnosis services within the context of antenatal care.

Six key themes were identified in the analysis – three from the pregnant women’s perspectives and three from the HCWs’ perspectives. Observations at the Polyclinic confirmed the themes identified through the interviews, including challenges such as long waiting times, the positive relationships between women and the HCWs, complaints of hunger, discomfort and physical strain from extended waiting on hard benches and the burden on two nurses managing more than 30 women a day.

Pregnant women valued the integrated diagnostic services and respectful care provided by the nurses but faced hidden costs, including additional payments and difficulties caused by long waits.

Healthcare workers highlighted systemic challenges such as inadequate resources, incomplete care continuity and fragmented donor support. Frequent shortages of essential supplies, treatments and diagnostic tools affected service quality, while fragmented donor contributions and inconsistent funding disrupted care delivery, creating resource gaps and breaking continuity.

The study highlights that robust healthcare systems – not just infrastructure – are essential for effective care. The results align with evidence from the Lancet Commission on Diagnostics, which found that at the advanced level of primary health care, HIV (65%) and malaria (62%) tests were the most readily available. However, key investigations recommended by WHO for antenatal care had variable but generally low availability, including syphilis testing (49%), urine dipsticks (52%), haemoglobin testing (37%), blood glucose testing (32%) and ultrasound (12%).^1^ Using the WHO building blocks for health systems,^19^ which focus on leadership and governance, financing, service delivery, workforce, medical technologies and information systems, gaps in healthcare delivery can be identified and addressed. To facilitate the discussion, the experiences of the HCWs and pregnant women have been categorised according to these WHO building blocks. While integrated diagnosis aims to improve care delivery and health outcomes, achieving these outcomes requires cohesive systems and sustainable support.

### Leadership and governance

Effective leadership and governance are vital for functional healthcare systems. Developing multi-disease policies and integrated guidelines can improve service uptake, as evidenced by antenatal guidelines at Mabvuku Polyclinic. However, many LMICs lack integrated testing policies, leaving separate frameworks for different diseases.^20,21^ This gap hinders integration efforts. Weak policy operationalisation, common in resource-limited settings, leads to suboptimal implementation.^22^ At Mabvuku Polyclinic, the absence of a substantive leader has created oversight and efficiency challenges.

### Health workforce

Service delivery depends on a capable and adequately resourced workforce. The WHO emphasises that integration cannot be a substitute for addressing systemic resource gaps.^10^ Expanding workloads without staff increases undermines service quality and staff well-being.^23^ There are examples of interventions where HCWs have expressed concerns about increased workloads and pressure due to integration efforts.^24,25^ In LMICs, asking HCWs to take on additional tasks without added compensation is particularly challenging unless these tasks are redefined as routine services. At Mabvuku Polyclinic, chronic understaffing – operating at half capacity – overburdens HCWs, increasing risks of errors and burnout. Locum nurses provide temporary relief but disrupt continuity and quality due to their limited familiarity with the clinic’s needs. Moreover, frequent absences due to donor-funded workshops, although beneficial for skills development, exacerbate shortages, compromising care delivery.

Addressing workforce gaps, ensuring equitable task distribution, and aligning staff capacity with service needs are vital to supporting integration. Without these measures, integrated interventions risk imposing undue strain on already stretched health systems.

### Service delivery

Service delivery is a cornerstone of effective health systems, requiring workforce capacity, timeliness and positive interactions between healthcare providers and patients.^11,19^ Timely care improves health outcomes for urgent conditions and allows patients to focus on other priorities. However, integration can inadvertently lead to inefficiencies, such as longer wait times, which frustrate patients and lower service uptake.^9,26^

Delays are a major challenge for integrated services. Studies, for example from Zambia, show integration can lengthen wait times due to inefficient workflows.^27^ While extended consultations allow for addressing multiple conditions, patients may avoid raising additional concerns to reduce delays for others.^28^

At Mabvuku Polyclinic, severe staff shortages result in women waiting up to 8 h, exacerbated by uncomfortable conditions. Yet, the convenience of multi-disease testing is highly appreciated. Respectful and compassionate midwives mitigate some frustrations, fostering trust and positive experiences. However, the high patient-to-nurse ratio can lead to rushed care and missed issues, increasing maternal and neonatal risks.

The quality of patient-provider relationships significantly influences patients’ perceptions of care.^29^ Feeling respected by HCWs is fundamental to how patients rate their overall experience.^28^ Patients who perceive respect from HCWs are more likely to report positive healthcare experiences. Therefore, HCWs must receive training not only in clinical diagnosis but also in delivering integrated, patient-centred care.

Although provider-initiated testing has been effective in increasing testing uptake,^28^ it does not always improve patient experiences. Power imbalances between providers and patients can make patients feel coerced into accepting tests. For instance, 29% of the patients in a study reported difficulty refusing HIV testing by TB nurses, fearing negative repercussions.^24^

At Mabvuku Polyclinic, the women highly valued the midwives’ friendliness and compassionate care, which helped mitigate frustrations with long waits and procedural inefficiencies. This approach fosters trust and positive perceptions of care. However, the high patient-to-nurse ratio often leads to rushed consultations, increasing the risk of errors, which compromises care quality, potentially contributing to higher maternal and neonatal mortality rates.

### Health system financing

Funding is crucial for the success of integrated diagnostic interventions in LMICs. Many rely heavily on donor funding, but integration efforts can stall when certain conditions, such as non-communicable diseases, are not donor priorities.^30,31^ While diseases like HIV benefit from significant donor support, the lack of funding for other areas undermines care.

Poor coordination of external funding often leads to fragmented services, inefficiencies and demotivation among HCWs. In Uganda, for example, donor incentives during health campaigns caused HCWs to neglect routine services, jeopardising sustainability when incentives ceased.^32,33^

At Mabvuku Polyclinic, financial constraints result in persistent resource shortages and inefficiencies. Critical equipment, such as BP machines and laboratory analysers, is frequently unavailable. Patients face significant out-of-pocket costs for transport, food, and supplies, deterring care-seeking and creating confusion about the affordability of supposedly ‘free’ services. Despite multiple donors, fragmented funding and poor coordination exacerbate resource gaps and overburden staff, undermining morale and service delivery.

### Access to medical products and technologies

Access to commodities is a significant barrier to integrated services in many LMICs. Frequent shortages of essential consumables and malfunctioning equipment significantly hinder healthcare delivery.^34,35^ At Mabvuku Polyclinic, these issues are acute, with frequent shortages and malfunctions of essential items disrupting routine care. These shortages force HCWs to prioritise critical cases, compromising the efficiency and quality of care.

### Health information systems

High-quality evidence is crucial for effective integrated diagnostic interventions, requiring standardised reporting tools, such as templates and registers, to ensure accountability and comprehensive care.^3^ Systems that prioritise all targeted diseases equally – without favouring donor-specific conditions – enable health workers to address diverse patient needs effectively.

At Mabvuku Polyclinic, challenges like poor connectivity and logistical barriers undermine the potential of these systems. These issues disrupt patient referrals, hinder follow-up tracking, and delay progress monitoring, reducing efficiency. Addressing these gaps is vital for optimising health outcomes and operational efficiency.

## Limitations of the study

This qualitative study offers valuable insights, but has limitations. Its findings are not generalisable to other settings because of the specific context of the Mabvuku Polyclinic. However, the study’s primary goal was to provide transferable insights into integrated diagnostic services in maternal healthcare, which may inform similar contexts.

Another limitation is recall bias, as participants may not accurately remember all the details. To mitigate this, interviews were conducted shortly after health visits, and carefully designed questions minimised biases, such as acquiescence bias, ensuring a more accurate portrayal of participants’ experiences.

### Policy implications

The Mabvuku Polyclinic study highlights several critical policy areas to improve integrated maternal healthcare services as discussed hereunder.

#### Staffing and training

Policies must focus on recruiting and retaining skilled personnel to alleviate staff shortages and reduce waiting times. Continuous training should ensure HCWs stay updated on best practices and technologies.

#### Service integration

Policies should prioritise fully integrated diagnostic services within maternal care. Co-locating diagnostic tests can improve efficiency and patient satisfaction. Ensuring consistent access to functional equipment and medical supplies is crucial for seamless service delivery.

#### Leadership and governance

Effective management practices are essential for optimising resources, maintaining equipment and coordinating stakeholders. Policies should encourage collaboration among donors, government bodies and non-governmental organisations (NGOs) to reduce service fragmentation and align funding with healthcare priorities.

#### Patient-centred care

Policies must promote respectful, patient-focused practices by addressing barriers like hunger, comfort during waiting and ambulance costs. Ensuring affordable or free maternal care services is vital while tackling corruption and improving HCW compensation to prevent accessibility issues.

#### Data utilisation and monitoring

Strengthening health information systems is key to identifying gaps, monitoring patient outcomes and guiding decisions. Policies should promote data-driven approaches to improve care delivery and policy effectiveness.

## Conclusion

The challenges at Mabvuku Polyclinic emphasise the need for a comprehensive approach to strengthening the health system. Key priorities include addressing staff shortages, enhancing leadership, ensuring consistent funding and maintaining essential medical supplies. The experiences of both HCWs and patients illustrate the importance of cohesive, well-supported systems for better health outcomes and patient satisfaction. Mabvuku Polyclinic serves as a reminder that robust healthcare requires more than infrastructure; it demands a strong workforce, efficient management and reliable resources to meet community needs effectively.
